# Organophosphate Detection in Animal-Derived Foods Using a Modified Quick, Easy, Cheap, Effective, Rugged, and Safe Method with Liquid Chromatography–Mass Spectrometry

**DOI:** 10.3390/foods13162642

**Published:** 2024-08-22

**Authors:** Byung-Joon Kim, Seung-Hyun Yang, Hoon Choi

**Affiliations:** 1 Department of Agricultural Biotechnology, Research Institute of Agriculture and Life Sciences, Seoul National University, Seoul 08826, Republic of Korea; payton20@snu.ac.kr; 2 Healthcare Advanced Chemical Research Institute, Environmental Toxicology & Chemistry Center, Hwasun-gun 58141, Republic of Korea; yangpa5000@gmail.com; 3 Department of Life and Environmental Sciences, College of Agriculture and Food Sciences, Wonkwang University, Iksan 54538, Republic of Korea

**Keywords:** organophosphate, animal-derived food, LC–MS/MS, QuEChERS

## Abstract

Organophosphates are widely used in the livestock industry. In this study, we developed a method for detecting 27 organophosphate insecticides in animal-derived foods, including beef, pork, chicken, milk, and eggs, using liquid chromatography–tandem mass spectrometry. A modified QuEChERS method was optimized for sample pretreatment. A mixture of acetonitrile and acetone was used as the extraction solvent, and MgSO_4_ and NaCl were used as salts. Among the five different dispersive solid-phase extraction systems, MgSO_4_, primary secondary amines, and C18 were selected for purification because they had the highest recovery rates and least matrix effects. The matrix-dependent limit of quantitation was 0.0005–0.005 mg/kg, and the correlation coefficient of the matrix-matched calibration curve was >0.99, which was acceptable for quantifying residues below 0.01 mg/kg—the default maximum residue limit in a positive list system. The recovery efficiencies ranged from 71.9 to 110.5%, with standard deviations ranging from 0.2% to 12.5%, satisfying the SANTE guidelines. The established analytical method was used to monitor organophosphates in animal-derived foods obtained from a local market, and no pesticides were detected. With respect to industry standards, our proposed method is recommended for practical organophosphate detection in animal-derived foods.

## 1. Introduction

Pesticides and veterinary drugs are used to protect crops and animals, respectively, from pests and diseases. Although they offer crop and animal protection, their impact on human health and the environment is concerning due to their inherent toxicity [1,2]. Pesticides in livestock products result from livestock consuming contaminated feed, while veterinary drugs remain in livestock products due to their use during animal rearing [3,4]. Consequently, pesticides and veterinary drugs can be detected in livestock products through indirect contamination from feed and direct use on animals. Moreover, since several active substances can be used either as pesticides or veterinary drugs, determining whether a substance detected in livestock products originated from pesticides used on feed crops or veterinary drugs used on livestock is impossible. Veterinary drugs such as insecticides are used to protect animals from pests in the livestock industry. However, the misuse of pesticides and non-approved veterinary drugs can lead to the contamination and poisoning of food-producing animals, with over 30% of such cases attributed to pesticide poisoning [5]. Pesticides tend to accumulate in animals due to their lipophilic characteristics, and consumption of pesticide-contaminated feeds can be fatal to livestock [6,7,8]. Such misuse has led to global issues related to pesticide-contaminated meat and meat products [8,9]. These incidents have highlighted global concerns regarding pesticide toxicity [10]. Therefore, comprehensive management without distinguishing the source is required to control chemical substances such as pesticides and veterinary drugs in livestock products.

Organophosphates (OPs) are among the most used pesticides worldwide, with the USA and Europe accounting for over 50% of global usage [11,12]. The OPs inhibit the nervous system of pests, a mechanism that poses risks to the environment and animals [11,12]. Because of their high solubility in water, OPs can also endanger aquatic organisms [11]. OP residues in food products have raised significant human health concerns due to the detrimental effects of long-term exposure to OPs [11,12,13]. In Korea, only two OPs, phosmet and phoxim, have been approved as veterinary drugs, while maximum residue limits (MRLs) for 11 OPs—chlorfenvinphos, diazinon, ethion, isofenphos, mecarbam, methidathion, monocrotophos, phenthoate, phosalone, profenofos, and triazophos—have been established for livestock products due to the possibility of pesticide pollution from feeds [14].

Pesticide analysis, including sample preparation, is of growing importance owing to the need to detect multiple pesticide residues in livestock, and the development of effective pesticide analysis methods for livestock [4,6,7,8,15] is an area of active research. The QuEChERS (Quick, Easy, Cheap, Effective, Rugged, and Safe) method, described by Anastassiades, has been widely adopted in various fields, including pesticide analysis [6,10,16,17,18,19]. Compared with traditional pretreatment methods, QuEChERS offers reduced solvent usage, rapid pretreatment through combined extraction and partitioning steps, and simplicity because of the use of dispersive solid-phase extraction (d-SPE) instead of SPE cartridges or open columns. However, simplifying the pretreatment process reduces the ability to remove matrix interference, thereby necessitating compensation for this loss. Therefore, mass spectrometry (MS) is also widely used with the QuEChERS method because of its high sensitivity and resolution, making it suitable for multiresidue analysis [6,20]. Using QuEChERS coupled with MS is effective for analyzing pesticides in low-fat-content samples (<20%), such as fruits, vegetables, milk, and eggs, leading to the development of various QuEChERS methods [13,17,21,22]. However, additional extraction and purification steps are required for high-fat-content samples to extract lipophilic pesticides and remove lipid interference substances. Meanwhile, few studies of OP analysis have been reported in animal-derived foods. Kang et al. [6] simultaneously analyzed 66 pesticides, including 12 OPs, in animal-derived foods using GC–MS, while Talyer et al. [10] reported an analytical method for 159 pesticides, including 9 OPs, in liver, kidney, and muscle using LC–MS. Ko et al. [7] analyzed phorate and its metabolites in animal-derived foods using LC–MS, and Anjos et al. [15] reported a method for analyzing 7 OPs in milk using LC–MS.

The Republic of Korea announced the implementation of a positive list system (PLS) in 2011. Under the PLS, a 0.01 mg/kg limit is applied when no MRL exists [23]. In 2019, the PLS was extended to all agricultural products, including fruits and vegetables. By 2024, PLS will be applied to veterinary drugs in the Republic of Korea. Some pesticides are categorized as veterinary drugs, most of which are insecticides [14]. Moreover, because unintentional pesticide contamination of livestock products through feeds and intentional residues of veterinary drugs for livestock breeding are managed separately, an integrated method for analyzing OPs in animal-derived foods is required [14]. Therefore, in this study, we developed an OP screening method in animal-derived foods, including beef, pork, chicken, milk, and eggs, using LC–MS/MS in conjunction with the improved QuEChERS extraction method to achieve a suitable and effective analysis method that meets the MRL criteria.

## 2. Materials and Methods

### 2.1. Chemicals and Reagents

Anilofos (≥98.0%), azinphos-methyl (≥95.0%), cadusafos (≥98.0%), chlorfenvinphos (≥95.0%), diazinon (≥98.0%), ethion (≥95.0%), ethoprophos (≥95.0%), fenamiphos (≥98.0%), fenamiphos-sulfoxide (≥98.0%), fenamiphos-sulfone (97.83%), fosthiazate (≥98.0%), isofenphos (≥98.0%), mecarbam (≥98.0%), methidathion (≥95.0%), mevinphos (97.31%), monocrotophos (≥98.0%), phenthoate (≥98.0%), phosmet (≥98.0%), phosphamidon (≥90.0%), phoxim (97.72%), profenofos (≥95.0%), pyraclofos (99.5%), pyridaphenthion (98.51%), quinalphos (≥98.0%), tebupirimfos (≥95.0%), triazophos (≥98.0%), and vamidothion (97.40%) were purchased from Sigma-Aldrich (St. Louis, MO, USA), Santa Cruz Biotechnology (Dallas, TX, USA), and LGC standard (Teddington, Middlesex, UK). HPLC-grade acetonitrile (MeCN), acetone (ACE), methanol (MeOH), and distilled water were purchased from Fisher Scientific (Waltham, MA, USA). A QuEChERS extraction kit (4 g magnesium sulfate, MgSO_4_, and 1 g sodium chloride, NaCl) was purchased from Agilent Technologies (Santa Clara, CA, USA). Additionally, d-SPE substances for purification (MgSO_4_, primary secondary amines (PSA), C18, and graphitized carbon black (GCB)) were obtained from Restek (Bellefonte, PA, USA). Z-sep was purchased from Sigma-Aldrich. Formic acid and ammonium formate were purchased from Sigma-Aldrich (St. Louis, MO, USA). Stock standard solutions containing all OPs (1000 µg/L of each) were prepared in MeCN, diluted with the same solvent to prepare working solutions ranging from 0.1 to 500 µg/L, and stored at −20 °C in a freezer. The matrix-matched standard solutions with 0.02–100 µg/L were obtained by further diluting the working solutions with blank samples.

### 2.2. Preparation of Animal-Derived Foods

Animal-derived foods, including beef (sirloin), pork (pork belly), chicken (leg meat), milk, and eggs—which are the main parts consumed in the Republic of Korea—were obtained from local markets. The meat samples were ground using a meat grinder (MG516 Pro 1600, Kenwood, Havant, UK), and milk and eggs were homogenized (T25 Digital Ultra-Turrax, IKA-Werke GmbH & Co., Staufen, Germany). Homogenized samples were used to develop the analytical method.

### 2.3. Instrumental Conditions

A Shimadzu Nexera XR HPLC coupled with a Shimadzu LCMS-8045 mass spectrometer (Shimadzu Corp., Kyoto, Japan) was used for the instrumental analysis. UK-C18 (100 mm × 2.6 mm, i.d. 3 µm; Imtakt, Portland, OR, USA), Kinetex-C18 (100 mm × 4.6 mm, i.d. 2.6 µm; Phenomenex, Torrance, CA, USA), XBridge-C18 (100 mm × 2.1 mm, i.d. 3.5 µm; Waters, Dublin, Ireland), and Cardenza-C18 (150 mm × 2.0 mm, i.d. 3 µm; Imtakt, Portland, OR, USA) columns were used to optimize chromatogram separation. MeOH and distilled water were used as mobile solvents, each containing 0.1% formic acid and 10 mM ammonium formate (mobile phases A and B, respectively). The gradient began with 100% mobile phase B and was maintained for 2 min. The concentration of the mobile phase B was then decreased to 30% over 5 min and maintained for 5 min. The concentration of the mobile phase B was further decreased to 0% over 3 min and maintained for 6 min. After 3 min, mobile phase B was increased to 100% and maintained for 3 min. The total run time was 27 min, and the flow rate was 0.3 mL/min. The column oven temperature was 40 °C, and 2 µL of the solution was injected.

Multiple reaction monitoring (MRM) was used to quantify MS/MS. Electrospray ionization (ESI) positive mode was used for ionization, with an interface voltage of 4.0 kV. The desolvation line, interface, and heat block temperatures were 250, 300, and 400 °C, respectively. A nitrogen generator (Euroscience, Seongnam, Republic of Korea) was used to produce nitrogen as the nebulizing and drying gas, and argon (purity: 99.999%) was used as the collision gas. The heating and drying gas flow rates were both set to 10 L/min. The nebulizing gas flow was 3.0 L/min.

For MRM optimization, a 100 µg/L standard solution dissolved in ACE and MeCN was used. The precursor ion of each pesticide was first selected using the full scan mode with a mass-to-charge ratio (*m*/*z*) ranging from 50 to 500. The fragmentation of the precursor ion at various collision energies (CEs) was confirmed using a product ion scan. The quantifier and qualifier ions of the MRM transitions and their corresponding CEs were determined based on sensitivity and selectivity. Shimadzu Labsolution software (ver. 5.97) was used for data acquisition and processing. The ionization forms and MRM optimized for 27 OPs are shown in Table 1.

### 2.4. Extraction Solvent Comparison for the Pretreatment

The pretreatment method was modified based on QuEChERS to optimize the livestock analysis. Three types of extraction solvents, including ACE, MeCN, and ACE/MeCN (5:5, *v*/*v*), were used to compare extraction efficiency. Five grams of sample were fortified to 0.02 mg/kg with the standard solution and then extracted with 20 mL of the extraction solvent. The extract was partitioned using the original QuEChERS kit and analyzed by LC–MS/MS. This process was repeated three times for each extraction solvent.

### 2.5. Clean-Up Comparison for the Pretreatment

The clean-up method was optimized after the extraction solvent was established. Considering its clean-up efficiency, five d-SPEs—(A) d-SPE #1: 150 mg MgSO_4_ and 25 mg PSA; (B) d-SPE #2: 150 mg MgSO_4_, 25 mg PSA, and 25 mg C18; (C) d-SPE #3: 150 mg MgSO_4_ and 25 mg C18; (D) d-SPE #4: 150 mg MgSO_4_, 25 mg PSA, and 25 mg GCB; and (E) d-SPE #5: 150 mg MgSO_4_ and 25 mg Z-sep—were compared. Samples fortified at 0.02 mg/kg with standard solutions were pretreated according to the procedure described in Section 2.4, purified using each d-SPE method, and then analyzed with LC–MS/MS. This process was performed in triplicate.

### 2.6. Matrix Effects on Quantitation

To assess the purification efficiency of d-SPE, the matrix effect (ME) was calculated using the following equation: ME (%) = (S_matrix_ − S_solvent_)/S_solvent_ × 100, where S_matrix_ and S_solvent_ represent the slopes of the calibration curves obtained using matrix-matched standard solutions and working solutions in ACE and MeCN, respectively [9].

### 2.7. Method Validation

Based on the SANTE guidelines [24], the following parameters were validated to assess the performance of the established analytical method: selectivity, limit of quantification (LOQ), linearity, accuracy, and precision. The selectivity for OP analysis was evaluated by analyzing blank samples to check for the presence of interference peaks at the retention times of the OPs in the chromatogram. Instrumental LOQs (ILOQs), defined as the concentrations corresponding to a signal-to-noise ratio (S/N) of 10, were determined by analyzing aliquots of matrix-matched standard solutions ranging from 0.02 to 5 µg/L. Matrix-dependent LOQs (MLOQs) were determined as the lowest levels at which accuracy and precision are guaranteed based on the ILOQ, injection volume, extraction solvent volume, sample weight, and dilution in the analytical method [9]. Instrumental repeatability was verified by calculating the % RSD of the peak area after seven consecutive analyses of the matrix-matched standard solution with concentrations of 20 µg/L. The linearity was evaluated using the coefficients of determination (R^2^) derived from the linear regression of the response data across concentrations ranging from 1 to 100 µg/L. Recovery efficiency tests were conducted to determine the accuracy and precision of the analytical methods. The control samples were fortified at three different concentrations (0.01, 0.1, and 0.5 mg/kg) by spiking them with appropriate amounts of the working standard solutions, with five replicates at each fortification level. Recovery efficiency tests were conducted using the following sample preparation procedures: Five grams of each sample at three fortification levels were extracted with 20 mL of ACE/MeCN (5:5, *v*/*v*) by shaking for 3 min at 1300 rpm using a high-speed shaker (2010 Geno/Grinder, SPEX^®^ SamplePrep, Metuchen, NJ, USA). The extract was then combined with 4 g MgSO_4_ and 1 g NaCl and shaken for 1 min at 1300 rpm. After centrifugation for 5 min at 4000 rpm (Combi-408, Hanil Science Inc., Gimpo, Republic of Korea), a 1 mL aliquot of the supernatant was transferred into a d-SPE tube containing 150 mg of MgSO_4_, 25 mg of PSA, and 25 mg of C18. The tube was shaken for 1 min and then centrifuged for 5 min at 13,000 rpm in a microcentrifuge (M15R, Hanil Science Inc., Gimpo, Republic of Korea). The supernatant (400 µL) was mixed with MeCN (100 µL) and analyzed using LC–MS/MS.

### 2.8. Collection of Real Samples

After validation, a real sample analysis was conducted. Forty-eight livestock samples, including beef, pork, chicken breasts and legs, milk, and eggs, were purchased from local markets in Iksan City, Republic of Korea. The purchased items were analyzed using the validated method.

## 3. Results

### 3.1. Instrument Condition Optimization and Column Comparison

HPLC–MS/MS retention times, MRM transitions, and CEs were optimized for each OP and are shown in Table 1. Fenamiphos and its metabolites were analyzed in this study because the fenamiphos residue in the animal commodities was defined as the sum of fenamiphos and its sulfoxide and sulfone expressed as fenamiphos for compliance with MRLs in the Republic of Korea, the Codex Alimentarius Commission, and European Union [14,25,26]. The transition with the highest intensity was determined as the quantifier ion, while the transition with the next highest intensity was selected as the qualifier ion for identification. The ion ratio for the quantifier and qualifier ions of each OP in sample extracts varied by less than 20%, which is within the acceptable variation according to the SANTE guidelines [24].

**Figure 1:**
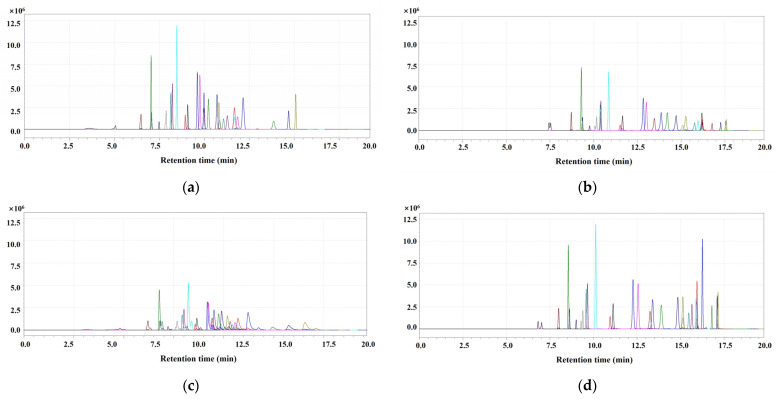
Comparison of LC–MS/MS chromatograms for organophosphates (200 µg/L) obtained from different columns: (**a**) UK-C18, (**b**) Kinetex-C18, (**c**) Xbridge-C18, and (**d**) Cardenza-C18.

The choice of the appropriate column for LC analysis depends on the nature of the analyte and specific properties such as orthogonality, column parameters, and analyte/stationary phase interactions [27]. Four different columns were compared to enhance selectivity and separation: (A) UK-C18, (B) Kinetex-C18, (C) Xbridge-C18, and (D) Cardenza-C18. Peak separation, sensitivity, and selectivity were considered for column selection. The UK-C18 column showed good sensitivity, but its separation was insufficient. Meanwhile, Xbridge-C18 and Kinetex-C18 showed good separation, but their sensitivity was low. The Cardenza-C18 column exhibited the best separation and sensitivity performance because it had the longest length and smallest diameter, which was subsequently utilized for the development of OP residue analysis (Figure 1).

### 3.2. Extraction Solvent Optimization

The OPs selected for this study exhibit varying degrees of polarity, with log *p* values ranging from −0.22 to 4.93 [28]. Therefore, ACE and MeCN, representative organic solvents used in multiresidue pesticide analysis, were considered extraction solvents [29].

ACE provided good extraction for most OPs but performed poorly for some nonpolar pesticides such as mecarbam (#13) and triazophos (#26). Additionally, ACE extracted large amounts of oils and fats from animal products, interfering with pesticide analysis and requiring extensive purification techniques [30].

MeCN also demonstrated good extraction efficiency for most OPs but had low extraction efficiency for certain pesticides, including azinphos-methyl (#2), chlorfenvinphos (#4), mevinphos (#15), monocrotophos (#16), and phosphamidon (#19). While eliminating the co-extraction of lipids is an advantage, challenges can still arise during MeCN extraction, particularly due to the formation of a fat layer between the aqueous and MeCN layers after extraction and partition steps, which can retain lipophilic pesticides and result in reduced recovery rates [31]. To circumvent these issues, employing mixed solvents for extraction is a viable solution. Since QuEChERS was first introduced by Anastassiades [16], various revised methods have been developed [4,6,7,13]. Many of these revised QuEChERS methods incorporate a mixture of different extraction solvents, often utilizing a combination of two solvents [19,32].

**Figure 2:**
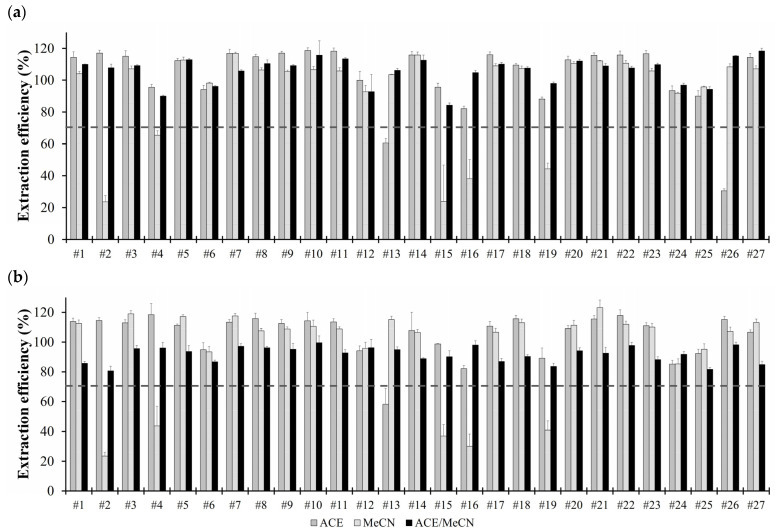
Extraction efficiencies of organophosphates in (**a**) eggs and (**b**) pork using various extraction solvents: acetone (ACE), acetonitrile (MeCN), and ACE/MeCN (5:5, *v*/*v*). The numbers (#1–#27) correspond to the organophosphates are listed in Table 1. The dashed lines indicate a recovery threshold of 70%.

The mixed solvent ACE/MeCN (5:5, *v*/*v*) combines the high extraction efficiency from fats in ACE with the effective extraction and partitioning enabled by salt addition in MeCN. This combination improved extraction outcomes, alleviated the issue of fatty layer formation, and ultimately enhanced the extraction efficiency of OPs (Figure 2). Therefore, this optimized extraction method was subsequently used in the clean-up procedure.

### 3.3. Clean-Up Comparison

**Figure 3:**
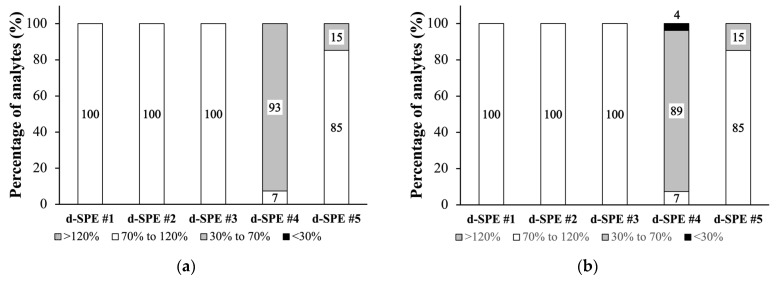
Recovery rate of organophosphates in (**a**) eggs and (**b**) pork following purification with different d-SPE systems: d-SPE #1 (150 mg MgSO_4_ and 25 mg PSA); d-SPE #2 (150 mg MgSO_4_, 25 mg PSA, and 25 mg C18); d-SPE #3 (150 mg MgSO_4_ and 25 mg C18); d-SPE #4 (150 mg MgSO_4_, 25 mg PSA, and 25 mg GCB); and d-SPE #5 (150 mg MgSO_4_ and 25 mg Z-sep).

Using ACE as the extraction solvent resulted in the extraction of larger amounts of lipids and other interfering substances compared to when using MeCN alone. Therefore, additional purification is necessary to remove co-extracted substances that adversely impact quantification. In this study, five d-SPE systems were compared for the clean-up of animal-derived food extracts with higher lipid content. All the d-SPE systems showed good recovery efficiency, except in the presence of GCB (d-SPE #4) and Z-sep (d-SPE #5), as shown in Figure 3.

In d-SPE #4 with GCB, overall recoveries were below 70%, except for isofenphos (71.8%) and phenthoate (91.6%) in eggs and monocrotophos (86.6%) and quinalphos (71.1%) in pork. GCB is commonly utilized for pigment adsorption but is known for its tendency to retain pesticides with planar structures, resulting in unsatisfactory recovery and poor precision [33,34]. Conversely, Z-sep is a silica support coated with zirconium dioxide and developed specifically for fatty matrix purification [35]. d-SPE #5 with Z-sep showed low recoveries only for fenamiphos-sulfoxide (63.4% in eggs; 60.8% in pork), fenamiphos-sulfone (51.4% in eggs; 57.1% in pork), isofenphos (60.8% in eggs; 65.9% in pork), and profenofos (62.9% in eggs; 56.3% in pork) among the OPs.

**Figure 4:**
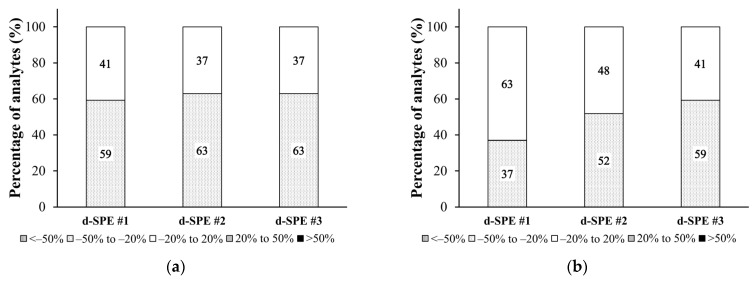

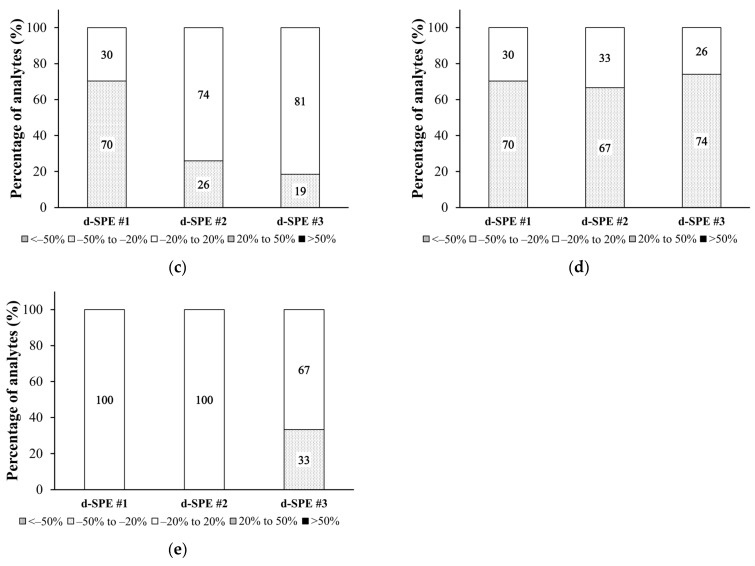
Impact of d-SPE on matrix effects of organophosphates in animal-derived foods. (**a**) beef, (**b**) pork, (**c**) chicken, (**d**) milk, and (**e**) eggs. The d-SPE systems are as follows. d-SPE #1: 150 mg MgSO_4_ and 25 mg PSA; d-SPE #2: 150 mg MgSO_4_, 25 mg PSA, and 25 mg C18; d-SPE #3: 150 mg MgSO_4_ and 25 mg C18.

To identify the most effective d-SPE method, we assessed the ME for each d-SPE method, excluding GCB and Z-sep due to their poor recoveries. The ME ranged from −44.1% to 12.5% across all matrices and d-SPE systems, which indicates a matrix-induced signal suppression. Of the three d-SPE methods, purification with d-SPE #2 demonstrated the lowest ME (Figure 4). Consequently, d-SPE #2, which includes MgSO_4_ (150 mg), PSA (25 mg), and C18 (25 mg), was chosen for purification to improve the removal of various interferents through its diverse component composition.

### 3.4. Method Validation of the Established Method

The analytical method developed, as reported in the previous sections, was fully validated according to the European SANTE/11312/2021 guidelines [24]. Blank samples were analyzed to ensure no interference peaks at the retention times of the OPs, confirming that the OPs were not subject to any interference. The ILOQ and MLOQ were 0.1–1 µg/L and 0.0005–0.005 mg/kg, respectively, which were sufficient to detect the 0.01 mg/kg level, the default MRL in PLS (Table 2). The RSDs of the peak areas at 20 µg/L for beef, pork, chicken, milk, and eggs were 0.5%, 1.2%, 1.8%, 0.6%, and 1.3%, respectively, indicating good instrumental repeatability. The R^2^ values for the regression equations were greater than 0.99 for the matrix-matched standard solutions in the range of 1–100 µg/L, demonstrating strong linearity and satisfying the criteria of >0.98.

The recoveries and repeatability of the developed method were assessed to evaluate the trueness and precision, respectively. As shown in Table 2, the recoveries of 27 OPs in animal-derived foods ranged from 71.9% to 110.5%, with RSDs from 0.2% to 12.5%, which satisfied the guideline criteria of recoveries between 70% and 120% and RSDs of ≤20% [24].

### 3.5. Real Sample Analysis

After validation, the analytical method that was developed was employed to monitor OPs in animal-derived foods and evaluate their applicability. Forty-eight animal-derived foods were purchased from the local market and analyzed three times for OP residues. No OPs were detected in any of the samples. The established analytical method was confirmed to be suitable for monitoring OPs in circulating livestock products. Additionally, the monitoring results indicated that the potential for residues from the illegal use of OPs in domestic livestock products is negligible.

## 4. Conclusions

QuEChERS has emerged as the most used method for pesticide analysis because of its efficiency. Researchers have recently adapted QuEChERS for livestock analysis, focusing on fat removal because pesticides, which are nonpolar, often interfere with fat removal. In this study, we established a simultaneous method for analyzing 27 OPs in animal-derived foods. Instrumental conditions were optimized, and all pesticides were analyzed in the positive mode of electrospray ionization, which is common in pesticide analysis. Gradient LC was employed to enhance separation and chromatogram reproducibility. Various C18 columns were compared to optimize the separation of the 27 OPs. Longer columns with smaller diameters improved the separation efficiency, although shorter columns reduced the total runtime and potentially compromised sensitivity and selectivity. Improved separation can lead to a reduction in MEs, enhancing the accuracy of pesticide analysis.

Three extraction solvents were evaluated, namely ACE, MeCN, and a mixture of ACE and MeCN. Both ACE and MeCN are effective pesticide extraction solvents, but the focus was on livestock characteristics rather than specific pesticide properties. ACE, which has a lower polarity index, tends to extract fats and lipids along with pesticides, leading to interference with selectivity. This results in lower extraction efficiencies for mecarbam and triazophos. MeCN, with a higher polarity index, extracted less fat and lipids but was less efficient at extracting several pesticides, including azinphos-methyl, chlorfenvinphos, mevinphos, monocrotophos, and phosphamidon. The combined solvent approach leverages the advantages of both ACE and MeCN. The ACE-MeCN mixture provided the most favorable extraction efficiency.

Although d-SPE may have slightly lower clean-up capabilities than SPE cartridges, the high selectivity of LC–MS/MS can compensate for this limitation. Five different d-SPE methods were compared, focusing on ME. Among the d-SPE methods, PSA and C18 exhibited the highest recovery rates, with ME of −44.1% to 12.5%. The lower the ME, the more applicable the method to various sample types. Therefore, the d-SPE system containing MgSO_4_, PSA, and C18 was determined as the optimal purification method.

The developed analytical method for OPs in animal-derived foods exhibited high sensitivity (MLOQ 0.0005–0.005 mg/kg), strong linearity of calibration curves (R^2^ >0.98), excellent instrumental repeatability (RSD < 2%), good trueness (recovery 71.9–110.5%), and high reproducibility (RSD < 13%). These attributes demonstrate its optimal configuration for high selectivity, sensitivity, accuracy, and precision. Furthermore, its application to real samples proved the method’s suitability for the routine analysis and monitoring of OPs in animal-derived foods by Korean authorities. Therefore, the method can be adopted as an official protocol for compliance with legislation and food safety management.

## Figures and Tables

**Table 1:** Multiple reaction monitoring (MRM) conditions for organophosphate insecticides using liquid chromatography coupled with tandem mass spectrometry (LC–MS/MS).

No.	Pesticide	Retention Time (min)	Molecular Weight	Ionization	Precursor Ion > Product Ion (CE ^1^)	
					Quantifier (*m*/*z*)	Qualifier (*m*/*z*)
1	Anilofos	15.1	367	[M + H]^+^	368.0 < 124.9 (−31)	368.0 < 170.9 (−22)
2	Azinphos-methyl	11.0	317	[M + H]^+^	318.0 < 125.0 (−20)	318.0 < 77.0 (−39)
3	Cadusafos	16.2	270	[M + H]^+^	271.0 < 158.9 (−15)	271.0 < 130.9 (−22)
4	Chlorfenvinphos	15.5; 15.9	358	[M + H]^+^	358.9 < 98.9 (−30)	358.9 < 169.9 (−40)
5	Diazinon	15.6	304	[M + H]^+^	305.0 < 153.0 (−25)	305.0 < 169.0 (−25)
6	Ethion	17.1	384	[M + H]^+^	385.0 < 198.9 (−10)	385.0 < 142.9 (−24)
7	Ethoprophos	13.3	242	[M + H]^+^	243.0 < 130.9 (−20)	243.0 < 96.9 (−22)
8	Fenamiphos	13.8	303	[M + H]^+^	304.0 < 216.9 (−25)	304.0 < 201.9 (−36)
9	Fenamiphos-sulfoxide	9.5	320	[M + H]^+^	320.0 < 232.9 (−19)	320.0 < 292.0 (−15)
10	Fenamiphos-sulfone	9.6	335	[M + H]^+^	336.0 < 265.9 (−20)	336.0 < 307.9 (−15)
11	Fosthiazate	10.1	283	[M + H]^+^	284.0 < 103.9 (−25)	284.0 < 227.9 (−10)
12	Isofenphos	15.9	345	[M + H]^+^	345.9 < 216.9 (−25)	345.9 < 245.0 (−12)
13	Mecarbam	13.2	329	[M + H]^+^	329.9 < 226.8 (−10)	329.9 < 96.9 (−37)
14	Methidathion	10.9	320	[M + H]^+^	320.9 < 145.0 (−10)	320.9 < 85.1 (−22)
15	Mevinphos	8.6; 9.0	224	[M + H]^+^	225.0 < 126.9 (−15)	225.0 < 192.9 (−8)
16	Monocrotophos	8.0	223	[M + H]^+^	223.9 < 193.0 (−8)	223.9 < 126.9 (−15)
17	Phenthoate	14.8	320	[M + H]^+^	321.0 < 247.0 (−11)	321.0 < 163.0 (−20)
18	Phosmet	11.1	317	[M + H]^+^	317.9 < 160.0 (−15)	317.9 < 77.0 (−20)
19	Phosphamidon	9.2; 9.3	299	[M + H]^+^	300.0 < 174.0 (−14)	300.0 < 226.9 (−13)
20	Phoxim	15.8	298	[M + H]^+^	298.9 < 77.0 (−30)	298.9 < 129.0 (−11)
21	Profenofos	16.7	374	[M + H]^+^	375.0 < 305.0 (−18)	375.0 < 347.0 (−13)
22	Pyraclofos	15.8	360	[M + H]^+^	361.0 < 256.9 (−22)	361.0 < 138.1 (−40)
23	Pyridaphenthion	12.2	340	[M + H]^+^	341.0 < 188.9 (−25)	341.0 < 205.0 (−24)
24	Quinalphos	15.1	298	[M + H]^+^	299.0 < 163.0 (−21)	299.0 < 147.0 (−21)
25	Tebupirimfos	17.1	318	[M + H]^+^	319.0 < 276.9 (−15)	319.0 < 231.0 (−24)
26	Triazophos	12.5	313	[M + H]^+^	314.0 < 162.0 (−20)	314.0 < 97.0 (−36)
27	Vamidothion	8.5	287	[M + H]^+^	288.0 < 145.9 (−10)	288.0 < 118.0 (−25)

^1^ CE, collision energy (unit, V).

**Table 2:** Recovery of organophosphate pesticides in animal-derived foods using modified QuEChERS with LC–MS/MS.

Compound	MLOQ (mg/kg)	Fortification (mg/kg)	Recovery (%, *n* = 5) ^1^				
			Beef	Pork	Chicken	Milk	Eggs
Anilofos	0.0005	0.01	72.6 ± 1.3	78.2 ± 3.1	75.9 ± 3.2	83.2 ± 2.6	88.1 ± 3.4
		0.1	92.6 ± 1.1	92.0 ± 1.4	92.2 ± 1.9	94.3 ± 1.3	96.4 ± 0.9
		0.5	93.0 ± 1.3	88.9 ± 0.8	89.0 ± 1.9	92.2 ± 1.0	92.9 ± 1.3
Azinphos -methyl	0.004	0.01	82.6 ± 7.0	90.3 ± 7.5	76.5 ± 3.1	86.3 ± 7.1	87.0 ± 5.0
		0.1	88.8 ± 2.6	91.1 ± 2.0	82.2 ± 1.9	96.6 ± 2.7	98.4 ± 2.2
		0.5	92.0 ± 2.2	88.0 ± 1.8	80.8 ± 1.7	95.9 ± 1.2	94.0 ± 0.7
Cadusafos	0.0005	0.01	79.0 ± 2.0	84.4 ± 1.4	77.4 ± 3.2	85.2 ± 1.9	85.8 ± 2.0
		0.1	87.6 ± 1.6	86.3 ± 0.9	90.1 ± 4.2	92.0 ± 1.4	93.5 ± 0.7
		0.5	89.1 ± 1.1	85.4 ± 1.1	89.2 ± 2.2	90.0 ± 2.0	89.8 ± 1.4
Chlorfenvinphos	0.001	0.01	72.1 ± 4.2	91.2 ± 4.3	73.4 ± 1.3	84.3 ± 3.7	94.4 ± 5.6
		0.1	91.8 ± 1.4	90.5 ± 1.4	87.0 ± 1.8	92.0 ± 0.9	94.5 ± 1.1
		0.5	92.9 ± 1.7	87.4 ± 1.0	83.7 ± 1.9	90.0 ± 2.0	92.0 ± 1.9
Diazinon	0.001	0.01	80.7 ± 2.3	91.5 ± 2.1	74.8 ± 3.0	84.3 ± 3.7	74.8 ± 3.0
		0.1	89.9 ± 1.0	86.4 ± 0.9	87.4 ± 1.5	91.9 ± 1.6	87.4 ± 1.5
		0.5	88.7 ± 1.5	84.3 ± 1.4	87.1 ± 2.8	90.8 ± 1.4	87.1 ± 2.8
Ethion	0.001	0.01	77.2 ± 3.1	103.1 ± 2.2	106.2 ± 1.3	100.8 ± 2.2	84.5 ± 3.0
		0.1	86.4 ± 1.4	99.4 ± 2.4	84.5 ± 3.2	91.0 ± 1.5	94.1 ± 1.4
		0.5	89.4 ± 0.9	96.7 ± 1.4	87.8 ± 4.2	86.5 ± 0.8	89.3 ± 2.3
Ethoprophos	0.0005	0.01	74.3 ± 3.9	79.6 ± 2.0	73.6 ± 3.7	73.1 ± 3.5	86.4 ± 2.1
		0.1	90.5 ± 1.4	89.9 ± 2.8	85.8 ± 1.0	92.5 ± 4.3	97.6 ± 1.3
		0.5	90.5 ± 1.7	87.1 ± 0.9	86.9 ± 3.8	89.5 ± 0.7	93.9 ± 0.7
Fenamiphos	0.0005	0.01	79.0 ± 1.2	75.0 ± 1.6	80.2 ± 2.0	84.1 ± 1.1	84.2 ± 2.1
		0.1	91.6 ± 1.4	90.7 ± 0.5	90.1 ± 3.9	93.3 ± 1.5	97.6 ± 1.3
		0.5	92.0 ± 1.3	90.3 ± 1.0	91.1 ± 2.8	92.0 ± 0.5	93.9 ± 0.7
Fenamiphos -sulfoxide	0.001	0.01	75.6 ± 4.8	81.2 ± 2.7	92.2 ± 6.3	73.1 ± 5.1	84.2 ± 1.1
		0.1	93.4 ± 1.9	89.8 ± 2.5	96.5 ± 1.6	91.8 ± 2.1	93.9 ± 0.7
		0.5	93.2 ± 1.7	90.7 ± 1.5	93.5 ± 2.0	90.2 ± 0.7	91.8 ± 0.8
Fenamiphos -sulfone	0.001	0.01	80.3 ± 4.9	80.9 ± 3.3	74.1 ± 1.9	96.3 ± 1.9	78.3 ± 2.0
		0.1	92.2 ± 1.8	92.2 ± 1.6	91.8 ± 2.2	92.2 ± 2.1	87.1 ± 2.2
		0.5	93.9 ± 2.1	91.1 ± 1.5	91.3 ± 2.4	90.3 ± 2.3	83.5 ± 1.3
Fosthiazate	0.0005	0.01	82.5 ± 1.0	79.4 ± 1.5	82.4 ± 2.8	78.7 ± 2.1	90.1 ± 2.3
		0.1	93.0 ± 1.0	91.7 ± 0.3	83.0 ± 1.2	93.7 ± 1.7	97.0 ± 1.0
		0.5	92.7 ± 1.0	90.3 ± 1.7	82.6 ± 1.2	91.7 ± 0.9	94.2 ± 0.4
Isofenphos	0.005	0.01	74.7 ± 3.8	90.9 ± 5.7	73.2 ± 3.6	94.9 ± 12.5	95.3 ± 8.2
		0.1	92.3 ± 4.8	85.4 ± 3.8	91.3 ± 4.9	102.3 ± 2.4	98.5 ± 1.5
		0.5	90.7 ± 2.1	82.6 ± 2.8	90.5 ± 2.8	96.0 ± 3.3	97.5 ± 4.0
Mecarbam	0.001	0.01	80.4 ± 2.3	78.8 ± 2.8	84.9 ± 3.9	85.8 ± 2.2	88.7 ± 2.1
		0.1	91.6 ± 1.4	89.2 ± 0.8	91.3 ± 1.2	92.4 ± 1.5	94.1 ± 1.1
		0.5	91.3 ± 1.5	89.0 ± 1.0	89.7 ± 2.3	90.9 ± 0.9	91.9 ± 1.2
Methidathion	0.001	0.01	82.9 ± 4.1	86.9 ± 3.1	88.5 ± 3.2	84.1 ± 5.4	84.2 ± 3.9
		0.1	88.3 ± 2.1	89.3 ± 1.0	92.3 ± 1.6	91.9 ± 1.2	95.8 ± 0.9
		0.5	87.9 ± 2.0	87.2 ± 1.2	90.2 ± 3.0	89.8 ± 1.1	91.4 ± 0.6
Mevinphos	0.001	0.01	72.6 ± 2.6	89.6 ± 1.4	89.3 ± 2.5	85.8 ± 3.6	89.3 ± 2.5
		0.1	92.6 ± 1.2	90.0 ± 1.3	91.6 ± 2.4	89.9 ± 2.6	91.6 ± 2.4
		0.5	93.7 ± 2.7	88.6 ± 0.8	92.8 ± 2.7	89.3 ± 1.3	92.8 ± 2.7
Monocrotophos	0.001	0.01	80.3 ± 5.9	79.9 ± 1.6	109.6 ± 6.8	85.5 ± 6.4	90.3 ± 6.7
		0.1	93.7 ± 1.4	83.1 ± 0.5	99.6 ± 2.3	92.4 ± 0.9	98.6 ± 0.5
		0.5	94.2 ± 2.1	79.9 ± 1.1	92.7 ± 4.1	88.1 ± 1.0	94.1 ± 1.0
Phenthoate	0.001	0.01	75.4 ± 3.8	83.1 ± 3.2	102.8 ± 2.1	101.5 ± 5.4	98.1 ± 4.3
		0.1	87.3 ± 1.1	84.5 ± 1.3	92.3 ± 1.4	96.4 ± 4.5	95.2 ± 2.0
		0.5	89.2 ± 1.8	84.4 ± 1.7	91.3 ± 1.8	93.5 ± 2.4	91.2 ± 1.3
Phosmet	0.0005	0.01	74.9 ± 3.5	79.9 ± 2.1	86.4 ± 2.1	87.1 ± 3.7	94.3 ± 3.2
		0.1	89.0 ± 1.7	88.0 ± 0.5	89.5 ± 3.5	90.6 ± 1.4	93.4 ± 0.4
		0.5	90.1 ± 1.6	87.8 ± 1.7	90.5 ± 1.8	89.1 ± 1.0	90.1 ± 0.8
Phosphamidon	0.001	0.01	73.7 ± 4.1	73.8 ± 1.7	105.3 ± 6.5	92.2 ± 1.8	95.3 ± 3.0
		0.1	86.0 ± 1.6	91.6 ± 0.4	104.6 ± 2.6	91.2 ± 0.7	92.5 ± 1.6
		0.5	89.2 ± 5.1	91.4 ± 0.7	104.0 ± 3.0	90.9 ± 0.7	90.3 ± 1.0
Phoxim	0.001	0.01	73.1 ± 3.6	85.2 ± 1.5	102.8 ± 7.1	79.0 ± 1.6	81.6 ± 4.4
		0.1	92.7 ± 3.0	89.9 ± 1.2	94.9 ± 3.4	94.7 ± 1.0	97.5 ± 0.4
		0.5	94.4 ± 1.2	87.2 ± 0.9	93.1 ± 1.4	93.9 ± 1.2	92.7 ± 1.5
Profenofos	0.003	0.01	88.6 ± 4.6	105.3 ± 6.9	107.5 ± 5.0	96.5 ± 5.3	86.2 ± 6.2
		0.1	85.8 ± 1.8	98.2 ± 1.4	95.6 ± 2.4	89.0 ± 2.4	94.4 ± 1.8
		0.5	87.2 ± 1.5	95.9 ± 1.5	91.3 ± 0.9	87.9 ± 0.8	90.5 ± 1.2
Pyraclofos	0.001	0.01	92.1 ± 2.0	93.6 ± 4.3	89.1 ± 7.2	92.9 ± 4.1	93.5 ± 3.0
		0.1	91.0 ± 2.1	88.1 ± 0.4	89.3 ± 3.9	93.3 ± 2.6	94.2 ± 1.2
		0.5	90.1 ± 1.3	85.8 ± 1.1	88.7 ± 1.5	90.9 ± 1.7	90.5 ± 1.0
Pyridaphenthion	0.001	0.01	83.5 ± 3.3	71.9 ± 3.5	82.2 ± 3.2	85.2 ± 2.6	85.5 ± 1.4
		0.1	91.5 ± 1.8	89.5 ± 0.8	89.0 ± 1.7	91.8 ± 1.2	94.1 ± 1.0
		0.5	91.6 ± 1.4	88.7 ± 0.5	89.1 ± 1.4	90.7 ± 0.7	91.9 ± 0.6
Quinalphos	0.001	0.01	75.4 ± 2.2	88.8 ± 2.5	85.3 ± 3.6	73.2 ± 3.3	78.1 ± 6.2
		0.1	93.3 ± 1.8	91.5 ± 1.7	95.8 ± 3.1	97.9 ± 3.5	96.5 ± 1.0
		0.5	92.9 ± 0.6	88.4 ± 0.9	91.6 ± 3.0	94.3 ± 1.4	93.3 ± 0.7
Tebupirimfos	0.001	0.01	83.2 ± 3.2	84.0 ± 5.6	108.7 ± 3.6	104.7 ± 4.9	109.1 ± 3.9
		0.1	84.9 ± 2.2	93.2 ± 1.3	89.1 ± 1.4	88.6 ± 1.5	88.1 ± 2.7
		0.5	85.0 ± 1.6	92.7 ± 1.9	85.8 ± 3.7	85.5 ± 2.0	83.3 ± 2.3
Triazophos	0.0005	0.01	72.5 ± 1.7	73.1 ± 1.3	81.7 ± 4.7	110.5 ± 5.3	78.4 ± 1.9
		0.1	92.5 ± 1.8	91.3 ± 0.6	93.8 ± 2.4	97.4 ± 1.6	93.4 ± 0.2
		0.5	93.0 ± 1.2	90.4 ± 0.5	91.9 ± 1.0	93.5 ± 0.7	92.5 ± 0.3
Vamidothion	0.0005	0.01	76.5 ± 3.9	74.2 ± 1.0	74.8 ± 1.1	76.2 ± 3.5	74.8 ± 1.1
		0.1	93.7 ± 1.4	91.1 ± 0.8	96.9 ± 1.2	92.4 ± 0.9	96.9 ± 1.2
		0.5	94.2 ± 2.1	89.1 ± 0.4	95.0 ± 0.9	88.1 ± 1.0	94.0 ± 0.6

^1^ mean ± standard deviation.

## Data Availability

The original contributions presented in the study are included in the article, further inquiries can be directed to the corresponding authors.

## References

[1] Choi H., Moon J.K., Kim J.H. (2013). Assessment of the exposure of workers to the insecticide imidacloprid during application on various field crops by a hand-held power sprayer. J. Agric. Food Chem..

[2] Li S., Zhang Q., Chen M., Zhang X., Liu P. (2020). Determination of veterinary drug residues in food of animal origin: Sample preparation methods and analytical techniques. J. Liq. Chromatogr. Relat. Technol..

[3] Food and Agriculture Organization of the United Nations (2016). Submission and Evaluation of Pesticide Residues Data for the Estimation of 430 Maximum Residue Levels in Food and Feed.

[4] Hamamoto K., Iwatsuki K., Akama R., Koike R. (2017). Rapid multi-residue determination of pesticides in livestock muscle and liver tissue via modified QuEChERS sample preparation and LC–MS/MS. Food Addit. Contam. Part A.

[5] Guitart R., Croubels S., Caloni F., Sachana M., Davanzo F., Vandenbroucke V., Berny P. (2010). Animal poisoning in Europe. Part 1: Farm livestock and poultry. Vet. J..

[6] Kang H.S., Kim M., Kim E.J., Choe W.-J. (2020). Determination of 66 pesticide residues in livestock products using QuEChERS and GC–MS/MS. Food Sci. Biotechnol..

[7] Ko A.-Y., Kim H., Jang J., Lee E.H., Ju Y., Noh M., Kim S., Park S.-W., Chang M.-I., Rhee G.-S. (2015). Development of an official analytical method for determination of phorate and its metabolites in livestock using LC–MS/MS. J. Food Hyg. Saf..

[8] Osesua A., Omoniyi F. (2022). Determination of pesticide residues in muscle and organs of cow, camel and goat in birnin kebbi, kebbi state, Nigeria. Int. J. Environ. Sci..

[9] Yang S.-H., Choi H. (2022). Simultaneous determination of nereistoxin insecticides in foods of animal origins by combining pH-dependent reversible partitioning with hydrophilic interaction chromatography–mass spectrometry. Sci. Rep..

[10] Taylor M.J., Giela A., Sharp E.A., Senior C.C., Vyas D.S. (2019). A rapid multi-class, multi-residue UHPLC–MS/MS method for the simultaneous determination of anticoagulant rodenticides, pesticides and veterinary medicines in wild animals, pets and livestock. Anal. Methods.

[11] Bhattu M., Verma M., Kathuria D. (2021). Recent advancements in the detection of organophosphate pesticides: A review. Anal. Methods.

[12] Kaushal J., Khatri M., Arya S.K. (2021). A treatise on organophophate pesticide pollution: Current strategies and advancements in their environmental degradation and elimination. Ecotoxicol. Environ. Saf..

[13] Collimore W.A., Bent G.-A. (2020). A newly modified QuEChERS method for the analysis of organochlorine and organophosphate pesticide residues in fruits and vegetables. Environ. Monit. Assess..

[14] Ministry of Food and Drug Safety (2024). Food Code.

[15] Dos Anjos M.R., Castro I.M.D., Souza M.D.L.M.D., de Lima V.V., de Aquino-Neto F.R. (2016). Multiresidue method for simultaneous analysis of aflatoxin M1, avermectins, organophosphate pesticides and milbemycin in milk by ultra-performance liquid chromatography coupled to tandem mass spectrometry. Food Addit. Contam. Part A.

[16] Anastassiades M., Lehotay S.J., Štajnbaher D., Schenck F.J. (2003). Fast and easy multiresidue method employing acetonitrile extraction/partitioning and “dispersive solid-phase extraction” for the determination of pesticide residues in produce. J. AOAC Int..

[17] Lehotay S.J., Son K.A., Kwon H., Koesukwiwat U., Fu W., Mastovska K., Hoh E., Leepipatpiboon N. (2010). Comparison of QuEChERS sample preparation methods for the analysis of pesticide residues in fruits and vegetables. J. Chromatogr. A.

[18] Morris B.D., Schriner R.B. (2015). Development of an automated column solid-phase extraction cleanup of QuEChERS extracts, using a zirconia-based sorbent, for pesticide residue analyses by LC–MS/MS. J. Agric. Food Chem..

[19] Steinbach P., Schwack W. (2014). Comparison of different solid-phase-extraction cartridges for a fatty acid cleanup of the ethyl acetate/cyclohexane based multi-pesticide residue method EN 12393. J. Chromatogr. A.

[20] Li C., Chu S., Tan S., Yin X., Jiang Y., Dai X., Gong X., Fang X., Tian D. (2021). Towards higher sensitivity of mass spectrometry: A perspective from the mass analyzers. Front. Chem..

[21] Choi B.S., Lee D.U., Kim W.S., Park C.W., Choe W.J., Moon M.J. (2023). Simultaneous screening of 322 residual pesticides in fruits and vegetables using GC-MS/MS and deterministic health risk assessments. Foods.

[22] Lawal A., Wong R.C.S., Tan G.H., Abdulra’uf L.B., Alsharif A.M.A. (2018). Recent modifications and validation of QuEChERS–dSPE coupled to LC–MS and GC–MS instruments for determination of pesticide/agrochemical residues in fruits and vegetables: Review. J. Chromatogr. Sci..

[23] Yun M.S., Choi H. (2023). Uptake of fungicide fluopyram from soil by scallions during greenhouse cultivation. Foods.

[24] European Commission (2021). Analytical Quality Control and Method Validation Procedures for Pesticide Residues Analysis in Food and Feed.

[25] CODEX Alimentarius Pesticide Index. https://www.fao.org/fao-who-codexalimentarius/codex-texts/dbs/pestres/pesticide-detail/en/?p_id=85.

[26] European Commission Pesticide Residue(s) and Maximum Residue Levels (mg/kg). https://ec.europa.eu/food/plant/pesticides/eu-pesticides-database/start/screen/mrls/details?lg_code=EN&pest_res_id_list=95&product_id_list=.

[27] Græsbøll R., Nielsen N.J., Christensen J.H. (2014). Using the hydrophobic subtraction model to choose orthogonal columns for online comprehensive two-dimensional liquid chromatography. J. Chromatogr. A.

[28] Turner J.A. (2018). The Pesticide Manual: A World Compendium.

[29] Maštovská K., Lehotay S.J. (2004). Evaluation of common organic solvents for gas chromatographic analysis and stability of multiclass pesticide residues. J. Chromatogr. A.

[30] Panseri S., Biondi P.A., Vigo D., Communod R., Chiesa L.M., Muzzaluop I. (2013). Occurrence of organochlorine pesticides residues in animal feed and fatty bovine tissue. Food Industry.

[31] Chamkasem N., Ollis L.W., Harmon T., Lee S., Mercer G. (2013). Analysis of 136 pesticides in avocado using a modified QuEChERS method with LC–MS/MS and GC–MS/MS. J. Agric. Food Chem..

[32] Wong J.W., Zhang K., Tech K., Hayward D.G., Krynitsky A.J., Cassias I., Schenck F.J., Banerjee K., Dasgupta S., Brown D. (2010). Multiresidue pesticide analysis of ginseng powders using acetonitrile- or acetone-based extraction, solid-phase extraction cleanup, and gas chromatography–mass spectrometry/selective ion monitoring (GC–MS/SIM) or –tandem mass spectrometry (GC–MS/MS). J. Agric. Food Chem..

[33] Koesukwiwat U., Sanguankaew K., Leepipatpiboon N. (2008). Rapid determination of phenoxy acid residues in rice by modified QuEChERS extraction and liquid chromatography–tandem mass spectrometry. Anal. Chim. Acta.

[34] Saito-Shida S., Nemoto S., Akiyama H. (2021). Multiresidue method for determining multiclass acidic pesticides in agricultural foods by liquid chromatography–tandem mass spectrometry. Anal. Methods.

[35] Belarbi S., Vivier M., Zaghouani W., De Sloovere A., Agasse V., Cardinael P. (2021). Comparison of different d-SPE sorbent performances based on quick, easy, cheap, effective, rugged, and safe (QuEChERS) methodology for multiresidue pesticide analyses in rapeseeds. Molecules.

